# Diffraction-limited axial scanning in thick biological tissue with an aberration-correcting adaptive lens

**DOI:** 10.1038/s41598-019-45993-4

**Published:** 2019-07-02

**Authors:** Katrin Philipp, Florian Lemke, Stefan Scholz, Ulrike Wallrabe, Matthias C. Wapler, Nektarios Koukourakis, Jürgen W. Czarske

**Affiliations:** 1 Technische Universität Dresden, Laboratory for Measurement and Sensor System Technique, Helmholtzstraße 18, 01069 Dresden, Germany; 2 University of Freiburg, Laboratory for Microactuators, Department of Microsystems Engineering-IMTEK, Georges-Köhler-Allee 102, 79110 Freiburg, Germany; 3 Helmholtz Centre for Environmental Research UFZ, Department of Bioanalytical Ecotoxicology, Leipzig, Germany

**Keywords:** Optical manipulation and tweezers, Adaptive optics, Optoelectronic devices and components, Confocal microscopy, Confocal microscopy

## Abstract

Diffraction-limited deep focusing into biological tissue is challenging due to aberrations that lead to a broadening of the focal spot. The diffraction limit can be restored by employing aberration correction for example with a deformable mirror. However, this results in a bulky setup due to the required beam folding. We propose a bi-actuator adaptive lens that simultaneously enables axial scanning and the correction of specimen-induced spherical aberrations with a compact setup. Using the bi-actuator lens in a confocal microscope, we show diffraction-limited axial scanning up to 340 μm deep inside a phantom specimen. The application of this technique to *in vivo* measurements of zebrafish embryos with reporter-gene-driven fluorescence in a thyroid gland reveals substructures of the thyroid follicles, indicating that the bi-actuator adaptive lens is a meaningful supplement to the existing adaptive optics toolset.

## Introduction

**Figure 1:**
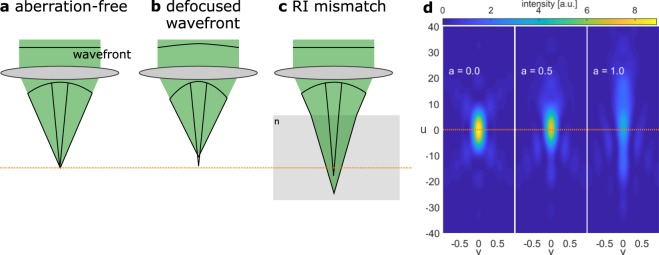
(**a**) Illustration of a diffraction-limited focus. (**b**) Illuminating a lens with a defocused wavefront (instead of a plane wave) leads to spherical aberrations. (**c**) A mismatch of the refraction indices (RIs) of the specimen and the surrounding medium results in spherical aberrations inside the specimen. (**d**) The calculated diffraction-limited focal spot without spherical aberrations (*a* = 0), shown in normalized optical coordinates *u* (axial) and *v* (radial). With an increasing amount *a* of spherical aberrations, the focal spot broadens in the axial and radial directions, and the maximum intensity decreases. The orange lines denote the nominal focal plane.

The inertia-free tunability of adaptive lenses leads to their application for the axial scaning in many microscopic techniques such as confocal microscopy^1–4^, two-photon microscopy^5,6^, structured illumination microscopy^7–10^, light sheet microscopy^11,12^ and standard wide-field microscopy^13–15^. However, these adaptive optical systems can only be optimized for one specific focal length of the adaptive lens. Thus, a diffraction-limited focal spot as illustrated in Fig. 1a is only achieved when the adaptive lens operates at the design focal length of the optical system. Tuning the adaptive lens introduces spherical aberrations that lead to a broadening of the focal spot as depicted in Fig. 1b. In microscopic applications, this focal spot broadening corresponds to rapid decreases in the lateral and in particular the axial resolution when the adaptive lens operates outside the design focal length^3,16^. To address this problem, adaptive lenses with advanced optical designs were developed to minimize the spherical aberrations at each focal length^17,18^. While these approaches greatly enhance the imaging quality of the adaptive lens, they still have only one degree of freedom. Therefore, these lenses cannot actively compensate aberrations that are induced by the interplay of the optical system components, such as lens illumination with a defocused wavefront as depicted in Fig. 1b.

Another spatial resolution limitation in microscopy is spherical aberrations induced by the specimens themselves due to refractive index mismatch (see Fig. 1c), which leads to deteriorating imaging quality with increasing penetration depth into a specimen. Spatial light modulators and deformable mirrors have been applied for aberration correction^19–23^ and scattering compensation^24–27^. While these adaptive-optics-based techniques enabled imaging of biological specimens with remarkable resolution and depth, they all have specific challenges: Using spatial light modulators in reflection mode^23^ or deformable mirrors^19^ requires beam folding and thus leads to bulky optical setups and hampers their assembly into existing optical systems such as commercial microscopes. In contrast, transmissive spatial light modulators can be integrated into optical systems in a collinear geometry. However, spatial light modulators suffer from a reduced transmission efficiency as they are polarization-selective, and typically only one diffraction order is used. Further, applying spatial light modulators or deformable mirrors for both aberration correction and axial scanning is possible; however, the tuning range of their refractive power is usually lower than that of adaptive lenses, and their implementation as a Fresnel lens results in a decrease in optical quality^28^. Additionally, their high number of degrees of freedom requires complex control or calibration strategies.

However, some applications, e.g., measurements of semi-transparent specimens with diameters in the low millimetre range such as zebrafish embryos, do not require sophisticated aberration correction and scattering compensation with a high number of degrees of freedom, as specimen-induced spherical aberrations are the key limitation of axial resolution: Zebrafish embryos and larvae are used as a model organism in developmental biology and toxicology^29^. In particular, transgenic models are highly attractive, since they allow the visualization of specific structures of interest using reporter-gene-driven fluorescence. Until approximately 6–8 days after fertilization, they are almost transparent. Thus, scattering only plays a minor role due to the high mean free path in a semi-transparent specimen. Hence, unwanted contributions from scattered photons can be efficiently suppressed by the pinhole of a confocal microscope^30^ up to penetration depths of several hundreds of microns into biological tissues^31^. Since the diameter of zebrafish embryos is typically on the order of 1 mm, specimen-induced spherical aberrations are the key limitation for spatial resolution. In order to achieve an acceptable imaging quality, thick biological specimens are usually elaborately prepared for optical microscopy, including slicing^32^ or clearing^33^. To supersede the need for extensive specimen preparation, a device with a reduced number of degrees of freedom that supports both axial scanning over a large tuning range and active spherical aberration compensation can be used to restore diffraction-limited axial scanning.

Adaptive lenses that extend their axial scanning capability by multiple additional degrees of freedom have great potential for both aberration correction and axial scanning over an extended specimen depth. The multi-actuator lens developed by Bonora *et al*.^21,34^ supports aberration correction up to the forth order of Zernike polynomials, and its refractive power tuning range is only approximately 0.5 dpt and therefore is not suitable for extended depth-resolved measurements in a specimen. In contrast, the segmented electrode lenses developed by Galstian *et al*.^35^ support a tuning range between 10 dpt and almost 200 dpt. As they were originally developed to be used with endoscopes^36,37^, their clear aperture is only 0.5 mm^35^; thus, these adaptive lenses are not suitable for optical microscopes. A special case of adaptive lenses with more degrees of freedom is the approach employed by Strauch *et al*.^38,39^, which uses a voltage signal to excite resonant oscillations on the surface of custom-built^38^ and commercial^39^ liquid lenses. While this approach supports a high number of degrees of freedom, it does not allow for the quasi-static operation that is required for sensor-less correction of specimen-induced aberrations. Further, these adaptive lenses with multiple degrees of freedom can be seen as a special case of transmissive spatial light modulators and as such easily achieve the requirements for diffraction-limited axial scanning in a semitransparent specimen with extended depth.

In contrast, Mishra *et al*.^40,41^ developed an adaptive lens with two degrees of freedom by combining fluidic and electrostatic tuning methods. However, the minimally supported refractive power is well above 50 dpt. Therefore, additional elements are required to include these lenses in an optical microscope (in order to operate the other elements like microscope objectives as designed). Further, the tuning range of the focal length is rather limited. In contrast, Fuh *et al*.^42^ achieved a large defocusing tuning range with limited success in spherical aberration correction by adopting a biconvex lens employing two thin polyvinyl chloride membranes whose thicknesses can be varied by the applied pressure.

In this paper, we propose a minimalistic approach for diffraction-limited axial scanning deep inside biological specimens using a bi-actuator adaptive lens with only two degrees of freedom – axial displacement and induced spherical aberrations. To demonstrate the potential of our approach, we use the adaptive lens in a confocal microscope. The optical sectioning capability of the confocal microscope efficiently suppresses out-of-focus scattering, leaving spherical aberration as the main contributor to the specimen-induced aberration. Using the bi-actuator adaptive lens, we simultaneously realize axial scanning and a reduction in spherical aberration for each focus position, leading to diffraction-limited foci deep in the biological specimen.

## Results

### Adaptive confocal microscope with spherical aberration correction

**Figure 2:**
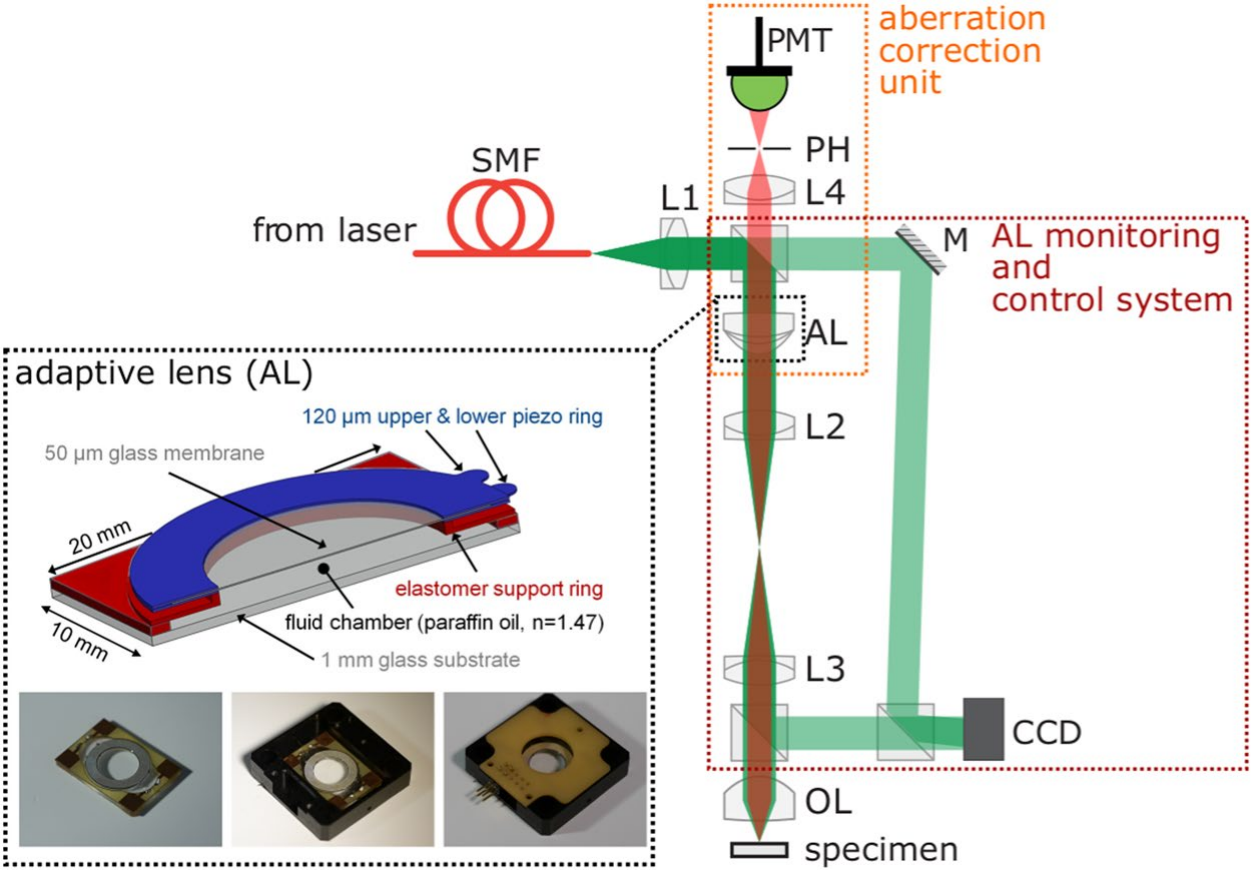
Illustration of a confocal microscope with axial scanning and an aberration correction unit. For clarity, only the fluorescence ray path is shown. Inset: Adaptive lens principle and photographs of the adaptive lens. SMF: single mode fibre. L: lens. OL: objective lens. AL; adaptive lens. M; mirror. PH: pinhole. PMT: detector. CCD: camera.

The setup of our custom-built adaptive confocal microscope is illustrated in Fig. 2. As the adaptive lens is employed both for axial scanning and spherical aberration compensation, the placement of the adaptive lens in the optical setup is crucial: To ensure axial scanning without inducing additional aberration, the planes of the adaptive lens and the objective lens (OL) of the microscope have to be mapped onto each other by a 4 f configuration. Otherwise, the two-fold propagation through the system with opposing directions will lead to additional defocusing aberrations at the position of the pinhole, compromising the axial resolution of the microscope^3,43^. Additionally, the adaptive lens has to be in the conjugate focal plane of the lens L4 before the detection pinhole to enable *direct* aberration sensing and compensation. The microscope is extended by an additional Mach-Zehnder interferometer for phase measurements and digital holography, whereby an off-axis configuration is used. A Zernike decomposition of the reconstructed phase is conducted, and the coefficients of the defocus and spherical aberration terms,

1 $${Z}_{{\rm{defocus}}}(r)=\sqrt{3}(2{r}^{2}-1),$$

2 $${Z}_{{\rm{SA}}}(r)=\sqrt{5}(6{r}^{4}-6{r}^{2}+1),$$

respectively, with the normalized radial coordinate *r* are used to control the adaptive lens.

Our adaptive lens is an advanced version of the lens introduced in^44^ and the active part of the lens consists of a 50 μm-thin glass membrane glued between two piezo rings (Fig. 1, inset). The high stiffness of the glass membrane allows for a higher actuation speed and opens up the possibility to shape the lens surface with more degrees of freedom compared to more flexible membrane materials, such as PDMS. The piezo-glass sandwich seals a fluid chamber that is filled with transparent paraffin oil. The two piezo actuator rings constitute two degrees of freedom for deforming the glass membrane by tuning the voltages *V*_1_ and *V*_2_ of the actuators. By applying opposite voltages to the upper and lower piezo ring, one ring expands while the other ring contracts which leads to an approximately spherical deformation of the glass membrane. When we actuate both piezo rings with the same positive voltage, both rings contract leading to a hyperbolical and thus aspherical deformation. Using a combination of this two actuation modes, we can control the focal power ${P}_{{\rm{AL}}}=\frac{1}{{f}_{{\rm{AL}}}}$ of the lens and also its spherical behaviour; see^45,46^ for a description how to actuate the lens at a specific defocus and spherical aberration. To compensate the creeping and rate-dependent hysteresis effects of the piezoelectric actuators, we implement a control system for the operation of the adaptive lens^45^.

### Spherical aberration sensing and correction with an adaptive lens

We adapt the protocol first introduced by M. Booth *et al*.^47^ for correction of specimen-induced aberrations in a confocal microscope. The aberration correction unit of the adaptive microscope consists of the adaptive lens, a pinhole and a PMT as illustrated in Fig. 2.

We assume a specimen that introduces a spherical aberration *aZ*_SA_(*r*) into the wavefront, where *a* is the Zernike coefficient of the spherical aberrations and *Z*_SA_(*r*) is the corresponding Zernike polynomial according Eq. (2). The range of the spherical aberration supported by the adaptive lens is denoted by the Zernike coefficient *b*_off_ that corresponds to the offset spherical aberration induced by the adaptive lens. The half width of the coefficient range is denoted by Δ*b*_max_. Therefore, the lens can induce arbitrary spherical aberrations *bZ*_SA_ with *b* = *b*_off_ + Δ*b* and −Δ*b*_max_ ≤ Δ*b* ≤ Δ*b*_max_. Note that *b*_off_, Δ*b* and Δ*b*_max_ are all functions of the refractive power *P*_AL_ of the adaptive lens; to enhance the presentation clarity, we do not explicitly denote this dependence.

For the compensation of the specimen-induced spherical aberration *aZ*_SA_, the adaptive lens is now used to deliberately induce aberrations of (*b* ± *β*)*Z*_SA_, where the parameter *β* is selected by the user. The difference in the signals *W*_±_ obtained behind the detection pinhole serves as the response curve to determine the corresponding Zernike coefficient of the wavefront.

The illumination point spread functions of a confocal microscope with inserted bias aberrations (*b* ± *β*)*Z*_SA_(*r*) are

3 $${H}_{\pm }({\rm{\Delta }}b;\upsilon ,\varphi )={| {\mathcal F} \{\exp (ia{Z}_{{\rm{SA}}}(r)+i({b}_{{\rm{off}}}+{\rm{\Delta }}b){Z}_{{\rm{SA}}}(r)\pm i\beta {Z}_{{\rm{SA}}}(r)+i\frac{u{r}^{2}}{2})\}|}^{2}$$

with a specimen-induced aberration *aZ*_SA_(*r*) and normalized axial and radial coordinates *u* and *v*, respectively, defined by $u=\frac{8\pi n}{\lambda }{\sin }^{2}(\alpha /\mathrm{2)}\cdot z$ and $\upsilon =\frac{2\pi }{\lambda }n\,\sin (\alpha )\cdot r$. Here, *α* is the angle with respect to the optical axis, and *r* and *z* are the radial and axial coordinates in real space, respectively. When considering a fluorescent sheet lying perpendicular to the optical axis as the specimen and assuming equal excitation and emission wavelengths, the detected signal after the pinhole is

4 $${W}_{\pm }({\rm{\Delta }}b)={\int }_{0}^{2\pi }\,{\int }_{0}^{{v}_{p}}({H}_{\pm }(\Delta b)\ast {H}_{\pm }(\Delta b))\upsilon {\rm{d}}\upsilon {\rm{d}}\varphi $$

where * denotes the correlation operator and *v*_*p*_ is the radius of the pinhole. The output signal of the modal wavefront sensor is obtained by the difference between the signals with negatively and positively aberrated wavefronts Δ*W*(Δ*b*) = *W*_+_(Δ*b*) − *W*_−_(Δ*b*). The measurement (and correction) of the Zernike coefficient *a* induced by the specimen is realized iteratively using

5 $${\rm{\Delta }}{b}_{n+1}={\rm{\Delta }}{b}_{n}+\gamma {\rm{\Delta }}W({\rm{\Delta }}{b}_{n}),$$

with the initial aberration correction coefficient *c*_0_ = 0 and the gain *γ* that is selected by the user depending on the desired convergence rate and supported Zernike coefficient range.

### Adaptive lens characteristics

**Figure 3:**
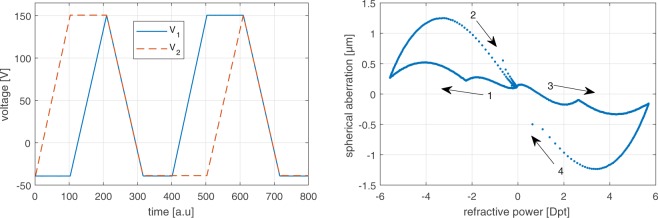
(**a**) Typical voltage trajectories for the actuation of the adaptive lens, illustrating the full possible operation region of the refractive power and spherical aberration tunability. The voltages *V*_1_ and *V*_2_ denote the actuation voltages of the two piezo actuators of the adaptive lens. (**b**) Spherical aberrations induced by the adaptive lens in relation to refractive power. The arrows and corresponding numbers illustrate the temporal evolution.

Figure 3b shows an example response of an adaptive lens when actuated with the voltages *V*_1_ and *V*_2_ of the piezo actuators depicted in Fig. 3a. The spherical aberration over the refractive power characteristics shown in Fig. 3b can be interpreted as the envelope of the supported operation region. The adaptive lens shows a pre-deflection due to internal strain caused by the manufacturing process, and a meta-stable jump occurs, where the lens flips from the counter side to the preferred stable pre-deflected side. The curve of spherical aberration vs. defocus suggests that the lens can compensate spherical aberrations induced by refractive index mismatch, but the lens does not allow zero aberration over the full focal power range. As a result, the optical design of the microscope has to be adapted to fully exploit the scanning range of the adaptive lens. Our approach to accomplish this task is to induce a positive spherical bias aberration in the optical system by choosing a front lens with a design wavelength of 488 nm, while the actual operation wavelength is 532 nm. A cover glass is used for the negative refractive powers of the adaptive lens in order to shift the spherical aberrations induced by the optical system towards those at positive refractive powers of the adaptive lens.

### Diffraction-limited axial scanning in free space

To demonstrate diffraction-limited axial scanning in free space, we use a mirror as a specimen. We measure the axial response of the confocal microscope, i.e., the intensity measured behind the pinhole as a function of the axial mirror position *z*. The full width half maximum (FWHM) of the axial response serves as a figure of merit for the axial resolution.

**Figure 4:**
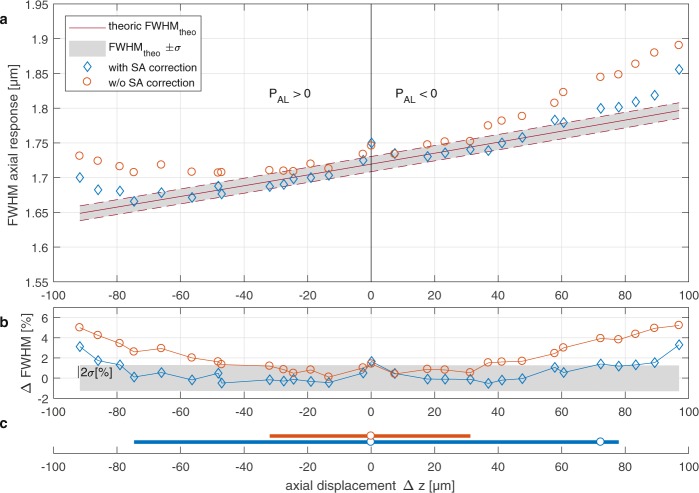
(**a**) FWHM of the axial responses of a scanned focus in air with and without spherical aberration correction as a function of the axial displacement of the focal position. A positive refractive power *P*_AL_ corresponds to a negative focal displacement Δ*z*, and vice versa. As a reference, the theoretical and minimum FWHM values obtained at a given focal length of the adaptive lens are shown. (**b**) Relative deviation of the measured FWHM from the diffraction limit. The grey area denotes ±2*σ* thresholds. (**c**) Regions in which diffraction-limited axial scanning is achieved. The white circles denote points where the diffraction limit is not achieved.

In the diffraction-limited case, the theoretical axial resolution^48^ of a confocal microscope under the assumption of an infinitely thin specimen is given by

6 $${\rm{FWHM}}({P}_{{\rm{AL}}})=\frac{0.67\lambda }{n-\sqrt{{n}^{2}-{{\rm{NA}}}^{2}({P}_{{\rm{AL}}})}}$$

where *n* is the refractive index of the surrounding medium, and the effective numerical aperture NA(*P*_AL_) changes with the refractive power *P*_AL_ of the adaptive lens. The effective numerical aperture is a combined effect of the adaptive lens (AL) and the objective lens (OL), which are mapped onto conjugate planes by a 4 f lens system as illustrated in Fig. 2. Using the thin lens approximation, the effective refractive power that defines the effective numerical aperture is *P*_eff_ = *P*_AL_ + *P*_OL_. Increasing the effective numerical aperture leads to an axial displacement Δ*z* of the focal spot of the confocal microscope in the opposing direction. To account for the misalignment of the 4 f system and the initial refractive power of the adaptive lens, we determine the effective numerical aperture by measuring the distance between the focal spot and the surface of the objective lens using a motorized stage. As a consequence, the theoretical FWHM increases almost linearly with the axial displacement Δ*z* of the focal spot, with values between 1.65 μm and 1.80 μm over an axial scanning range of 189 μm as shown in Fig. 4a. The error margin is calculated on the basis of the measurement uncertainty of the effective numerical aperture without actuating the adaptive lens, leading to a value of approximately 22 nm (full width 2*σ*) over the measured axial range (see Fig. 4a).

For an axial scanning system containing a conventional adaptive lens without the capability of tuning spherical aberrations, we expect the axial resolution to be worse than the resolution of the diffraction-limited case due to the objective lens (OL) operating outside its designed operation mode (e.g., illumination with a converging or diverging beam instead of a plane wave), and often spherical aberrations are induced by the adaptive lens itself. We actuate our adaptive lens with approximately equal actuation voltages for both piezo actuators to mimic a conventional adaptive lens (i.e., *V*_1_ = *V*_2_). As expected, the axial resolution degrades by up to 5%, and the deviation is largest for high absolute refractive powers of the adaptive lens. As a result, only 78 μm out of the total axial scanning range of 189 μm are diffraction-limited, where all FWHMs with less than a 2*σ* deviation are considered diffraction-limited as shown in Fig. 4c (red bar).

If we actuate the adaptive lens with spherical aberration compensation, the axial resolution improves as illustrated in Fig. 4. Here, the focal spot is axially diffraction-limited over an axial tuning range of more than 150 μm, as shown in Fig. 4c (blue bar), excluding Δ*z* = 0 and Δ*z* = 72 μm. The resolution is above FWHM_theory_ + 2*σ* at Δ*z* = 72 μm, which can be attributed to the fact that only 95.45% of Gaussian-distributed data points are expected to lay within the ±2*σ* interval about the median value. In contrast, the diffraction limit is not achieved for zero refractive power of the adaptive lens due to the low tuning range of the spherical aberration at this point; see Fig. 3. Compared with the uncorrected case, the spherical aberration correction yields an increase in the diffraction-limited scanning range by more than a factor of two.

### Specimen-induced aberration correction with a phantom specimen

**Figure 5:**
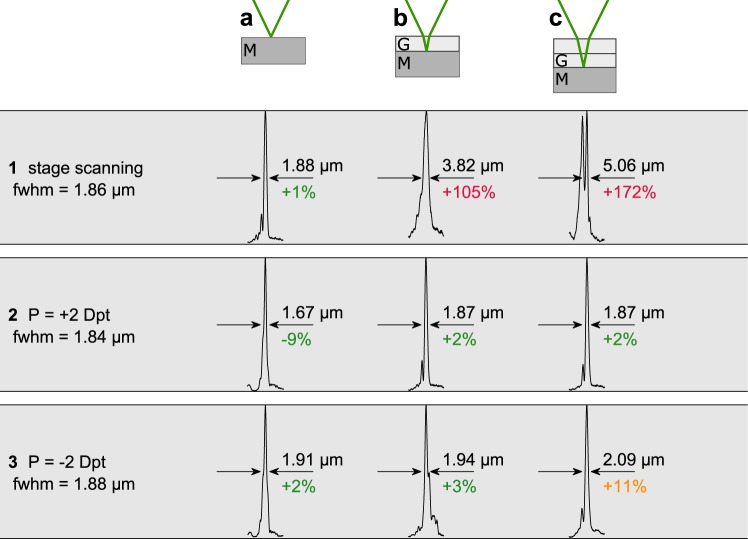
Phantom specimen consisting of one mirror (**a**) and one mirror and one (**b**) and two (**c**) cover glasses. Line (1) Corresponding normalized axial response curves employing stage scanning without spherical aberration compensation. Line (2) Axial response obtained with aberration correction at a refractive power of 2 dpt. Line (3) Axial response with aberration correction at −2 dpt. The theoretically calculated FWHMs for the diffraction-limited cases are denoted in the left column. The measured FWHM are stated for the corresponding axial response curves. The deviations of the measured FWHM from the diffraction limit are expressed in percentages. M: mirror. G: cover glass.

In order to test the spherical aberration correction capability of our adaptive microscope for specimen-induced aberrations, we use three types of phantom specimens to mimic a refractive index mismatch. The phantom specimens consist of a mirror and up to two cover glasses (see the header row in Fig. 5) with a thickness of 170 μm and a refractive index of *n*_BK7_ = 1.52 at *λ* = 532 nm (BK7, Schott AG). Consequently, measurements at depths *d*_BK7_ of 170 μm and 340 μm in the phantom specimen are conducted. As the index of refraction of zebrafish embryos is approximately *n*_embryo_ = 1.35 according to^49^ and thus significantly deviates from the refractive index of BK7, we use the optical path length OPL = *nd* to apply the observations of this section to zebrafish embryos. As the optical path length is invariant to the surrounding medium, the depths corresponding to BK7 inside the zebrafish embryos are $\frac{{n}_{{\rm{BK7}}}}{{n}_{{\rm{embryo}}}}{d}_{{\rm{BK7}}}$, which yields propagation depths of 189 μm and 378 μm for one and two cover glasses, respectively. As the thyroid of zebrafish embryos is not expected to be positioned much more than 200 μm beneath the surface^32^, our goal is to demonstrate diffraction-limited axial scanning up to this depth.

As a reference, we record the axial response of the confocal microscope with our adaptive lens in its default state at *V*_1_ = *V*_2_ = 0 for different positions of the mirror; see first row in Fig. 5. When using a mirror without cover glasses as the specimen, the axial resolution is limited by the numerical aperture NA = 0.6 of the objective lens according to Eq. (6), which yields FWHM = 1.86 μm. The FWHM of the measured axial response equals 1.88 μm, which deviates only by 1% from the theoretically obtained diffraction limit; see Fig. 5, cell **1a**. As expected, the FWHM of the axial response increases with increasing propagation depth by a factor of up to 2.7 (or an increase of 172%) due to the refractive index mismatch.

We demonstrate spherical aberration correction with an exemplary specimen at refractive powers of *P*_AL_ = ±2 dpt to ensure that the procedure works for both diverging and converging wavefronts when reaching the objective lens. The theoretical diffraction limits according to Eq. (6) change to 1.84 μm and 1.88 μm for *P*_AL_ = ±2 dpt, respectively, due to the influence of the refractive power of the adaptive lens on the effective numerical aperture of the optical system. The FWHMs obtained from the measured axial responses are in the expected region: 9% below or 2% above the theoretically obtained diffraction limit for *P*_AL_ = ±2 dpt, as shown in Fig. 5 (cells **2a** and **3a**). When using the phantom specimen consisting of one cover glass on top of the mirror, we do not expect the FWHM to change, as the effective numerical aperture remains constant. The FWHMs of the measured curves fulfil this expectation as they are only 2% to 3% above the theoretically obtained diffraction limit, as apparent from Fig. 5 (cells **2b** and **3b**). In the case of *P*_AL_ = + 2 dpt, this trend continues for the phantom specimen with two cover glasses on top of the mirror, and the measured FWHM is again only 2% above the diffraction limit. In the case of *P*_AL_ = −2 dpt, however, the FWHM obtained from the measured axial response is 11% higher than the diffraction limit; i.e., the focal spot is only near-diffraction-limited in this specific configuration (see cell **3c** in Fig. 5). As apparent from rows **b** and **c** in Fig. 5, the axial broadening of the focal spots 170 μm deep into the phantom specimen is reduced from 105% by factors of 50 and 35 to broadenings of 2% and 3% for refractive powers of *P*_AL_ = ±2 dpt, respectively, compared to the reference measurements with the stage scanning system. At a propagation depth of 340 μm into the specimen, the axial broadening is reduced from 172% by factors of 86 and 17 to 2% and 11% for refractive powers of *P*_AL_ = ±2 dpt, respectively, compared to the reference measurements with the stage scanning system.

### Correction of specimen-induced aberrations in *in vivo* measurements of zebrafish embryo thyroids

After the demonstration of diffraction-limited axial scanning inside a phantom specimen, we now use our adaptive confocal microscope to measure reporter-gene-driven fluorescence in the thyroid gland of a zebrafish embryo with and without spherical aberration correction. As shown in the previous section, diffraction-limited axial scanning is possible for phantom measurements up to at least 340 μm deep in BK7. This corresponds to a penetration depth of 378 μm in zebrafish embryos due to the invariance of the optical path length in the surrounding medium. As the diameter of zebrafish embryos typically does not exceed 1 mm and the thyroid is not located more than 200 μm beneath the surface^32^, we expect to achieve diffraction-limited or near-diffraction-limited focal spots by spherical aberration correction for measurements of the reporter-gene-driven fluorescence in the thyroid gland of zebrafish embryos.

**Figure 6:**
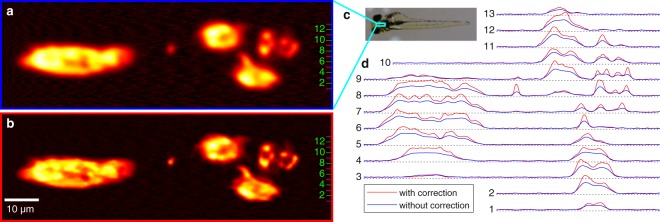
Zebrafish embryo at 110 hours post-fertilization with reporter-gene-driven fluorescence in the thyroid gland. Ventral views; anterior is on the left. Fluorescence images using the adaptive microscope without (**a**) and with (**b**) spherical aberration correction. The images are normalized separately to show the intensities using the full colour range. (**c**) The measured region is approximately marked in the standard wide-field image of the whole embryo. (**d**) The line profiles of the grey values of the non-normalized fluorescence images are taken at specific rows, where the positions of the lineouts are indicated by the green and black numbers in (**a**,**b**,**d**). The lineouts of the fluorescence images indicate that aberration correction leads to increased signal intensity and better optical sectioning. The dimensions are (100 × 308) m^2^ (**a**,**b**), approximately (1.5 × 5) mm^2^ (**c**) and (70 × 308) m^2^ (**d**).

In order to investigate the effect of our spherical aberration correction procedure on the measurement of zebrafish embryos, we acquire a fluorescence image of the thyroid without spherical aberration correction as a reference. We operate our microscope with a slight spherical aberration bias for a rough compensation of the refractive index mismatch. As a result, the reference image as shown in Fig. 6a is already pre-compensated for spherical aberration. To ensure that photobleaching and phototoxicity do not compromise our results, uncorrected images without applying the spherical aberration correction procedure are acquired before the images with the correction. The follicles of the thyroid are already distinctly perceivable in the pre-compensated reference image, while the substructure cannot be resolved in detail. In contrast, the substructure of the follicles is apparent in the image acquired with specimen-induced aberration correction, which allows a better understanding of the spatial distribution and extension of the thyroid follicles (Fig. 6b). The images are normalized separately to show the intensities using the full colour range.

The correction of specimen-induced aberrations also increases the contrast and overall signal strength, which is directly apparent from comparing the line profiles of the image with specimen-induced aberrations and the reference as shown in Fig. 6d. The maximum intensity increases by a factor of approximately two. The contrast is defined as the quotient of the standard deviation and mean value of the intensity values distinctly evaluated over the follicles. The contrast increases by various factors between 1.5 and 2.8 depending on the position and size of the evaluation window. The line profiles also highlight the appearance of follicle substructures when spherical aberration correction is applied; see e.g. the left side of the line profiles numbered 7, 8 and 9.

## Discussion

The main result of this paper is that our bi-actuator lens is capable of axial focal scanning while maintaining an axially diffraction-limited spot both in free space and deep inside specimens, even when employed in a multi-component imaging system such as a confocal microscope. This result is achieved by precisely controlling the lens shape, which allows the manipulation of spherical aberrations that are either induced by the specimen or by the optical system due to the axial scanning procedure. It should be noted that diffraction-limited axial scanning is restored with only two (and therefore the minimal possible number of) degrees of freedom.

As a result, the bi-actuator lens constitutes an ideal extension of the adaptive optics landscape: The adaptive lens is suited for applications that require axial scanning over an extended depth while restoring diffraction-limited foci inside a semi-transparent, quasi-homogeneous specimen. The bi-actuator lens is operated in transmission mode and is compatible with the standard 30 mm optical cage system. Therefore, the adaptive lens can be easily used to extend an existing microscope. On the other hand, deformable mirrors are the best choice for low-to-medium aberration correction in a non-homogeneous specimen with only little scattering. However, their operation is only possible in a reflection geometry, which results in the requirement of beam folding. Therefore, after some maturation, multi-actuator adaptive lenses are a suitable alternative for some of these applications. Highly scattering specimens, however, require a larger number of degrees of freedom that is currently only offered by spatial light modulators. However, this comes at the cost of reduced light efficiency due to diffraction and polarization selectivity, which is especially impactful in the detection path when investigating a fluorescent specimen. Therefore, the choice of using an approach based on (bi-actuator) adaptive lenses, deformable mirrors or spatial light modulators depends on the specific application.

In this paper, we focused on an application that benefits from using a bi-actuator adaptive-lens-based approach, namely fluorescence measurements deep inside zebrafish embryos. We applied the bi-actuator lens in a confocal microscope and therefore suppressed scattered photons through the pinhole. The bi-actuator lens was used for spherical aberration correction in zebrafish embryos with reporter-gene-driven fluorescence in the thyroid gland. This resulted in increased contrast and an enhanced fluorescence signal (Fig. 6b,d). Due to the improved optical sectioning, we were able to observe substructures of the thyroid follicles that were not visible without the spherical aberration correction.

In conclusion, the proposed bi-actuator adaptive lens enables a simple, compact and potentially low-cost method to extend an existing imaging system with the capability of diffraction-limited axial scanning. The presented approach leverages success from inertia-free, diffraction-limited axial scanning with adaptive lenses for spherical aberration compensation and can be employed in applications with high demands regarding optical quality and penetration depth. The presented approach is transferable to confocal techniques such as confocal Brillouin spectroscopy^50^, two/multi-photon microscopy with spherical aberration compensation^51^ and high-resolution laser machining.

Future work will focus on optimizing the characteristics of the adaptive lens (Fig. 3b) to allow zero spherical aberration over the full defocus range by employing a recently developed simulation technique^52^. This will enable diffraction-limited axial scanning in free space at both negative and positive refractive powers of the adaptive lens without the need to adapt the optical system accordingly.

## Materials and Methods

### Characterization of the adaptive lens surface

The adaptive lens was characterized by scanning the lens surface with a confocal distance sensor in combination with an xy stage while the lens was actuated using a function generator. The reconstructed time-dependent three-dimensional membrane shape was then used to obtain the defocus and spherical aberration Zernike coefficients of the resulting optical path difference (shown in Fig. 3) considering the refractive index of the adaptive lens fluid *n*_AL_. The refractive power of the adaptive lens was calculated from the defocus coefficient analogously to Eq. (7).

### Phase measurements and Zernike decomposition

The phase measurements were conducted with an off-axis Mach-Zehnder interferometer, whereby the hologram was recorded by a CCD camera (pco.pixelfly usb, 1392 × 1040 pixel, pixel size 6.5 μm, 14 bit dynamic range). The angular spectrum beam propagation method^53^ was used to determine the phase of the wavefront in the conjugate plane of the adaptive lens. A Zernike decomposition of the phase was conducted to extract the defocus and spherical aberration coefficients. The refractive power *P* of the adaptive lens was obtained from the defocus term of the measured wavefront using

7 $${P}_{{\rm{AL}}}=({n}_{{\rm{AL}}}-1)\frac{4\sqrt{3}\lambda {\alpha }_{2}^{0}}{{a}^{2}}$$

where *n*_AL_ = 1.48 is the refractive index of the lens fluid, *a* is the radius of the aperture used for the Zernike evaluation and ${\alpha }_{2}^{0}$ is the defocus Zernike coefficient. A derivation of Eq. (7) can be found in^45^. It should be noted that the combined effect of the adaptive lens and the two achromatic lenses of the relay system is measured (compare Fig. 2); thus, the control occurs due to the effective spherical aberration and defocus of these three lenses. Consequently, aberrations due to imperfect optical alignment along the optical axis are partly compensated as well.

### Control of spherical aberration and defocus induced by the adaptive lens

The creeping and hysteresis effects of the piezoelectric actuators make it necessary to control the actuation process. We implemented a control system based on wavefront measurements that also compensated influences from the environment such as temperature and pressure. The Zernike coefficients were determined as described in the previous paragraph. The initial actuation voltages were determined based on a previously recorded look-up table for actuation voltages, defocus and spherical aberration Zernike coefficients. The actuation voltages were then applied to the adaptive lens, and the Zernike coefficients of the wavefront were obtained. The actuation voltages were then refined iteratively based on the gradient of the look-up table.

### Zebrafish embryo preparation

Zebrafish of the strain tg:mCherry were originally provided by the University of Brussels^32^. The embryos were obtained as described by Fetter *et al*.^54^. Fish were cultured and used according to German and European animal protection standards and fish culture was approved by the Government of Saxony (Landesdirektion Leipzig, Aktenzeichen 759185.64). For imaging of the thyroid gland, embryos at 5 dpf were used. The embryos were anaesthetised with a tricaine solution (150 mg/L, TRIS 26 μmM, pH 7.5) and embedded in 3% methyl cellulose to stabilise the dorsoventral position.

### Fluorescence image acquisition of zebrafish embryos

The images were obtained by lateral scanning using a Newport XPS system. The *y* axis was used as the slow axis, and the *x* axis was used as the fast axis. The position along the *y* axis was moved stepwise in steps of 500 nm. The velocity of the *x* axis was set to 2 mm/s with an acceleration of 20 mm/s^2^. The acquisition rate of the Newport XPS system was set to 4 kHz, yielding a step width of approximately 500 nm after the acceleration process finished. The obtained scattered data were resampled on a regular grid with a pixel size of 500 × 500 nm^2^ using the *ScatteredInterpolant* class of MATLAB(R) using linear interpolation.

### Spherical aberration correction procedure in the measurements of zebrafish embryos

Since the mCherry dye is sensitive to photobleaching, minimizing the exposure time was a key priority when establishing the correction procedure. Consequently, we only measured the laterally integrated intensity for the iterative aberration correction using Eq. (5). We performed frame-wise aberration correction, because intermittent illumination causes less photobleaching than continuous illumination^55^.

## Data Availability

Experimental data are available from the corresponding author on reasonable request.
